# (2,7-Dimeth­oxy­naphthalen-1-yl)(2,4,6-trimethyl­phen­yl)methanone

**DOI:** 10.1107/S1600536811039225

**Published:** 2011-09-30

**Authors:** Toyokazu Muto, Kosuke Sasagawa, Akiko Okamoto, Hideaki Oike, Noriyuki Yonezawa

**Affiliations:** a Department of Organic and Polymer Materials Chemistry, Tokyo University of Agriculture & Technology, 2-24-16 Naka-machi, Koganei, Tokyo 184-8588, Japan

## Abstract

In the title compound, C_22_H_22_O_3_, the dihedral angle between the naphthalene ring system and the benzene ring is 82.93 (5)°. The bridging carbonyl C—C(=O)—C plane makes dihedral angles of 50.11 (6) and 46.87 (7)°, respectively, with the naphthalene ring system and the benzene ring. In the crystal, three types of weak inter­molecular C—H⋯O inter­actions are observed.

## Related literature

For electrophilic aromatic substitution of naphthalene derivatives, see: Okamoto & Yonezawa (2009 ▶); Okamoto *et al.* (2011 ▶). For the structures of closely related compounds, see: Muto *et al.* (2010 ▶); Watanabe *et al.* (2010 ▶, 2011 ▶).
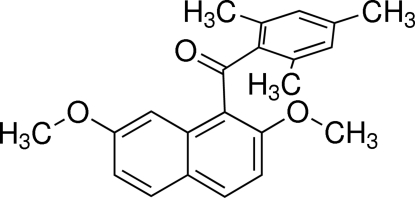


## Experimental

### 

#### Crystal data

C_22_H_22_O_3_
*M* *_r_* = 334.40
Monoclinic, 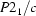

*a* = 10.5238 (4) Å
*b* = 12.2289 (4) Å
*c* = 15.0504 (5) Å
β = 111.340 (2)°
*V* = 1804.11 (11) Å^3^
*Z* = 4
Cu *K*α radiation
μ = 0.64 mm^−1^
*T* = 193 K
0.50 × 0.40 × 0.20 mm

#### Data collection

Rigaku R-AXIS RAPID diffractometer
Absorption correction: numerical (*NUMABS*; Higashi, 1999 ▶) *T*_min_ = 0.739, *T*_max_ = 0.882
32585 measured reflections
3295 independent reflections
2945 reflections with *I* > 2σ(*I*)
*R*_int_ = 0.023

#### Refinement

*R*[*F* ^2^ > 2σ(*F* ^2^)] = 0.039
*wR*(*F* ^2^) = 0.112
*S* = 1.08
3295 reflections
232 parameters
H-atom parameters constrained
Δρ_max_ = 0.26 e Å^−3^
Δρ_min_ = −0.25 e Å^−3^

### 

Data collection: *PROCESS-AUTO* (Rigaku, 1998 ▶); cell refinement: *PROCESS-AUTO*; data reduction: *CrystalStructure* (Rigaku/MSC, 2004 ▶); program(s) used to solve structure: *SIR2004* (Burla *et al.*, 2005 ▶); program(s) used to refine structure: *SHELXL97* (Sheldrick, 2008 ▶); molecular graphics: *ORTEPIII* (Burnett & Johnson, 1996 ▶); software used to prepare material for publication: *SHELXL97*.

## Supplementary Material

Crystal structure: contains datablock(s) I, global. DOI: 10.1107/S1600536811039225/zk2031sup1.cif

Structure factors: contains datablock(s) I. DOI: 10.1107/S1600536811039225/zk2031Isup2.hkl

Supplementary material file. DOI: 10.1107/S1600536811039225/zk2031Isup3.cml

Additional supplementary materials:  crystallographic information; 3D view; checkCIF report

## Figures and Tables

**Table 1:** Hydrogen-bond geometry (Å, °)

*D*—H⋯*A*	*D*—H	H⋯*A*	*D*⋯*A*	*D*—H⋯*A*
C4—H4⋯O1^i^	0.95	2.54	3.3756 (18)	147
C7—H7⋯O2^ii^	0.95	2.60	3.466 (2)	152
C18—H18*B*⋯O3^iii^	0.98	2.59	3.471 (2)	149

Symmetry codes: (i) 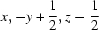; (ii) 
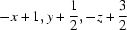
; (iii) 
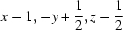
.

**Fig. 1.**
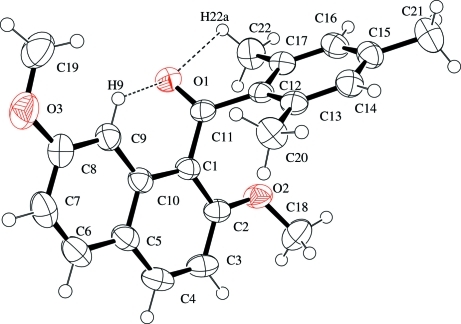
Molecular structure with displacement ellipsoids at 50% probability and two types of weak intramolecular C—H···O interactions.

**Fig. 2.**
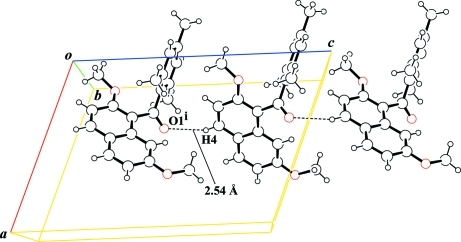
Intermolecular C4—H4···O1i interactions, viewed along the b axis [symmetry code: (i) x, -y + 1/2, z - 1/2].

**Fig. 3.**
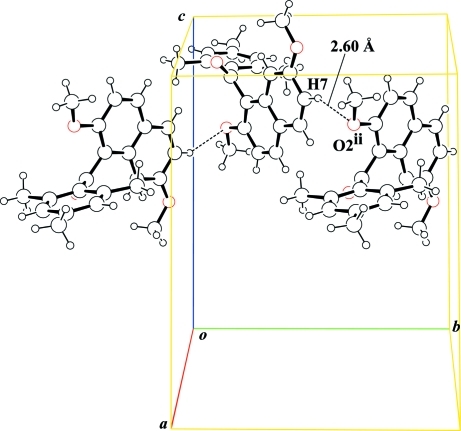
A packing diagram of the title compound, showing intermolecular C7—H7···O2 ii interactions [symmetry code: (ii) –x + 1, y + 1/2, -z + 3/2].

**Fig. 4.**
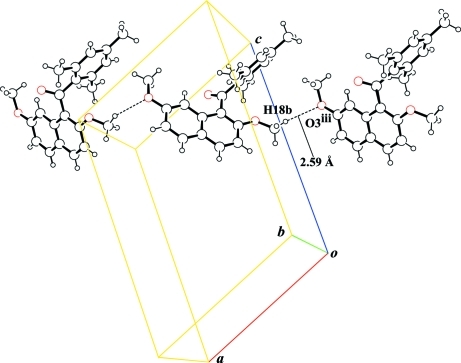
A packing diagram of the title compound, showing intermolecular C18—H18b···O3 iii interactions [symmetry code: (iii) x - 1, -y + 1/2, z - 1/2].

## supplementary crystallographic information

### Comment

In the course of our study on electrophilic aromatic acylation of 2,7-dimethoxynaphthalene, *peri*-arylcarbonylnaphthalene compounds have proven to be formed regioselectively with the aid of suitable acidic mediators (Okamoto & Yonezawa, 2009; Okamoto, Mitsui *et al.*, 2011). Recently, we have reported the crystal structures of several 1,8-diarylcarbonylated naphthalene analogues exemplified by 1,8-bis(4-methylbenzoyl)-2,7-dimethoxynaphthalene (Muto *et al.*, 2010). The arylcarbonyl groups at the 1,8-positions of the naphthalene rings in these compounds are connected in an almost perpendicular fashion. Besides, the crystal structures of arylcarbonylated naphthalene homologues, 1-monoarylcarbonylated naphthalene compounds and the *β*-isomers of 3-monoarylcarbonylated naphthalene compounds, have been also clarified such as (2,7-dimethoxynaphthalen-1-yl)(4-fluorophenyl)methanone (Watanabe *et al.*, 2011) and (3,6-dimethoxy-2-naphthyl)(4-fluorobenzoyl)methanone (Watanabe, Muto, Nagasawa *et al.*, 2010).

As a part of our continuing study on the molecular structures of these homologous molecules, the crystal structure of title compound, 1-monoarylcarbonylnaphthalene bearing three methyl groups on the arylcarbonyl group, is discussed in this report.

The molecular structure of the title compound is displayed in Fig. 1. The 2,4,6-trimethylphenyl group is out of the plane of the naphthalene ring. The dihedral angle between the best planes of the 2,4,6-trimethylphenyl ring (C12—C17) and the naphthalene ring (C1—C10) is 82.93 (5)°. The carbonyl group makes torsion angles of -130.97 (14) and -131.79 (13)°, respectively, with the naphthalene ring and the benzene ring [C2—C1—C11—O1 torsion angle = -130.97 (14)°; O1—C11—C12—C13 torsion angle = -131.79 (13)°]. In addition, two types of intramolecular C—H···O interactions are observed (C9—H9···O1 = 2.39 Å and C22—H22*a*···O1 = 2.51 Å; Fig. 1 and Table 1).

In the crystal structure, the molecular packing of the title compound is stabilized mainly by van der Waals interactions. The crystal packing is additionally stabilized by three types of C—H···O hydrogen bondings: Intermolecular C—H···O hydrogen bonding between the oxygen atom (O1) of the carbonyl group and one hydrogen atom (H4) of the naphthalene ring of the adjacent molecule is formed along the *c* axis (C4—H4···O1^i^; Fig. 2 and Table 1). There is also intermolecular C—H···O hydrogen bonding between the oxygen atom (O2) of 2-methoxy group and one hydrogen atom (H7) of the naphthalene ring of the adjacent molecule along the *b* axis (C7—H7···O2^ii^; Fig. 3 and Table 1). Furthermore, an intermolecular C—H···O hydrogen bonding between the oxygen atom (O3) of the 7-methoxy group and one hydrogen atom (H18*b*) of the 2-methoxy group of the adjacent molecule along the *ac* diagonal (C18—H18*b*···O3 ^iii^; Fig. 4 and Table 1) is observed.

### Experimental

To a 10 ml flask, 2,4,6-trimethylbenzoyl chloride (0.55 mmol, 100 mg), aluminium chloride (0.60 mmol, 80.0 mg) and methylene chloride (1.25 ml) were placed and stirred at 273 K. To the reaction mixture thus obtained, 2,7-dimethoxynaphthalene (0.50 mmol, 94.0 mg) was added. After the reaction mixture was stirred at 273 K for 6 h, it was poured into ice-cold water (10 ml). The aqueous layer was extracted with CHCl_3_ (10 ml × 3). The combined extracts were washed with 2 *M* aqueous NaOH followed by washing with brine. The organic layers thus obtained were dried over anhydrous MgSO_4_. The solvent was removed under reduced pressure to give cake. The crude product was purified by recrystallization from methanol (yield 56%). Colorless platelet single crystals suitable for X-ray diffraction were obtained by repeated crystallization from hexane and CHCl_3_.

### Refinement

All H atoms were found in a difference map and were subsequently refined as riding atoms, with C—H = 0.95 (aromatic) and 0.98 (methyl) Å, and with *U*_ĩso_(H) = 1.2 *U*_eq_(C).

### Crystal data

**Table:**

C_22_H_22_O_3_	*F*(000) = 712
*M**_r_* = 334.40	*D*_x_ = 1.231 Mg m^−^^3^
Monoclinic, *P*2_1_/*c*	Melting point = 408.0–410.0 K
Hall symbol: -P 2ybc	Cu *K*α radiation, λ = 1.54187 Å
*a* = 10.5238 (4) Å	Cell parameters from 15327 reflections
*b* = 12.2289 (4) Å	θ = 3.2–68.1°
*c* = 15.0504 (5) Å	µ = 0.64 mm^−^^1^
β = 111.340 (2)°	*T* = 193 K
*V* = 1804.11 (11) Å^3^	Block, colorless
*Z* = 4	0.50 × 0.40 × 0.20 mm

### Data collection

**Table:**

Rigaku R-AXIS RAPID diffractometer	3295 independent reflections
Radiation source: rotating anode	2945 reflections with *I* > 2σ(*I*)
graphite	*R*_int_ = 0.023
Detector resolution: 10.000 pixels mm^-1^	θ_max_ = 68.2°, θ_min_ = 4.5°
ω scans	*h* = −12→12
Absorption correction: numerical (*NUMABS*; Higashi, 1999)	*k* = −14→14
*T*_min_ = 0.739, *T*_max_ = 0.882	*l* = −18→18
32585 measured reflections	

### Refinement

**Table:**

Refinement on *F*^2^	Secondary atom site location: difference Fourier map
Least-squares matrix: full	Hydrogen site location: inferred from neighbouring sites
*R*[*F*^2^ > 2σ(*F*^2^)] = 0.039	H-atom parameters constrained
*wR*(*F*^2^) = 0.112	*w* = 1/[σ^2^(*F*_o_^2^) + (0.0568*P*)^2^ + 0.4166*P*] where *P* = (*F*_o_^2^ + 2*F*_c_^2^)/3
*S* = 1.08	(Δ/σ)_max_ < 0.001
3295 reflections	Δρ_max_ = 0.26 e Å^−^^3^
232 parameters	Δρ_min_ = −0.25 e Å^−^^3^
0 restraints	Extinction correction: *SHELXL97* (Sheldrick, 2008), Fc^*^=kFc[1+0.001xFc^2^λ^3^/sin(2θ)]^-1/4^
Primary atom site location: structure-invariant direct methods	Extinction coefficient: 0.0080 (5)

### Special details

**Table:**

Geometry. All e.s.d.'s (except the e.s.d. in the dihedral angle between two l.s. planes) are estimated using the full covariance matrix. The cell e.s.d.'s are taken into account individually in the estimation of e.s.d.'s in distances, angles and torsion angles; correlations between e.s.d.'s in cell parameters are only used when they are defined by crystal symmetry. An approximate (isotropic) treatment of cell e.s.d.'s is used for estimating e.s.d.'s involving l.s. planes.
Refinement. Refinement of *F*^2^ against ALL reflections. The weighted *R*-factor *wR* and goodness of fit *S* are based on *F*^2^, conventional *R*-factors *R* are based on *F*, with *F* set to zero for negative *F*^2^. The threshold expression of *F*^2^ > σ(*F*^2^) is used only for calculating *R*-factors(gt) *etc*. and is not relevant to the choice of reflections for refinement. *R*-factors based on *F*^2^ are statistically about twice as large as those based on *F*, and *R*- factors based on ALL data will be even larger.

### Fractional atomic coordinates and isotropic or equivalent isotropic displacement parameters (Å^2^)

**Table:**

	*x*	*y*	*z*	*U*_iso_*/*U*_eq_	
O1	0.37023 (10)	0.11774 (8)	0.92101 (7)	0.0486 (3)	
O2	0.12893 (10)	0.13899 (8)	0.66767 (6)	0.0484 (3)	
O3	0.68551 (11)	0.43082 (10)	1.02164 (8)	0.0629 (3)	
C1	0.30975 (12)	0.22890 (10)	0.78444 (9)	0.0358 (3)	
C2	0.23219 (13)	0.21417 (11)	0.68881 (9)	0.0400 (3)	
C3	0.26304 (15)	0.27038 (12)	0.61732 (10)	0.0481 (4)	
H3	0.2094	0.2590	0.5519	0.058*	
C4	0.37039 (16)	0.34113 (12)	0.64283 (10)	0.0497 (4)	
H4	0.3916	0.3780	0.5944	0.060*	
C5	0.45068 (14)	0.36101 (11)	0.73909 (10)	0.0438 (3)	
C6	0.55965 (15)	0.43751 (12)	0.76592 (12)	0.0539 (4)	
H6	0.5804	0.4755	0.7178	0.065*	
C7	0.63442 (15)	0.45735 (13)	0.85870 (13)	0.0565 (4)	
H7	0.7077	0.5081	0.8752	0.068*	
C8	0.60373 (14)	0.40273 (12)	0.93107 (11)	0.0487 (3)	
C9	0.50014 (13)	0.32777 (11)	0.90886 (9)	0.0415 (3)	
H9	0.4809	0.2915	0.9584	0.050*	
C10	0.42134 (12)	0.30417 (10)	0.81193 (9)	0.0379 (3)	
C11	0.27808 (13)	0.16488 (10)	0.85879 (8)	0.0358 (3)	
C12	0.13503 (13)	0.15946 (10)	0.85716 (8)	0.0353 (3)	
C13	0.05612 (13)	0.25478 (10)	0.84617 (9)	0.0385 (3)	
C14	−0.07551 (14)	0.24589 (11)	0.84654 (9)	0.0437 (3)	
H14	−0.1292	0.3102	0.8389	0.052*	
C15	−0.13086 (14)	0.14637 (12)	0.85775 (10)	0.0458 (3)	
C16	−0.05042 (14)	0.05397 (11)	0.87003 (10)	0.0462 (3)	
H16	−0.0867	−0.0146	0.8788	0.055*	
C17	0.08180 (14)	0.05783 (10)	0.86999 (9)	0.0406 (3)	
C18	0.02367 (17)	0.14400 (14)	0.57628 (12)	0.0638 (4)	
H18A	−0.0067	0.2199	0.5618	0.077*	
H18B	−0.0533	0.0986	0.5759	0.077*	
H18C	0.0583	0.1170	0.5281	0.077*	
C19	0.66243 (19)	0.37810 (16)	1.09841 (13)	0.0703 (5)	
H19A	0.6729	0.2989	1.0937	0.084*	
H19B	0.5698	0.3944	1.0955	0.084*	
H19C	0.7287	0.4046	1.1590	0.084*	
C20	0.11031 (16)	0.36753 (11)	0.83889 (12)	0.0504 (4)	
H20A	0.1250	0.3741	0.7784	0.061*	
H20B	0.1969	0.3789	0.8921	0.061*	
H20C	0.0441	0.4227	0.8414	0.061*	
C21	−0.27449 (16)	0.14021 (15)	0.85686 (14)	0.0639 (5)	
H21A	−0.2992	0.0636	0.8606	0.077*	
H21B	−0.3376	0.1726	0.7977	0.077*	
H21C	−0.2800	0.1805	0.9117	0.077*	
C22	0.16095 (16)	−0.04745 (12)	0.87986 (13)	0.0569 (4)	
H22A	0.2396	−0.0467	0.9401	0.068*	
H22B	0.1927	−0.0544	0.8265	0.068*	
H22C	0.1018	−0.1095	0.8795	0.068*	

### Atomic displacement parameters (Å^2^)

**Table:**

	*U*^11^	*U*^22^	*U*^33^	*U*^12^	*U*^13^	*U*^23^
O1	0.0445 (5)	0.0555 (6)	0.0429 (5)	0.0012 (4)	0.0126 (4)	0.0122 (4)
O2	0.0554 (6)	0.0534 (6)	0.0327 (5)	−0.0105 (4)	0.0115 (4)	−0.0034 (4)
O3	0.0491 (6)	0.0660 (7)	0.0622 (7)	−0.0138 (5)	0.0066 (5)	−0.0090 (5)
C1	0.0394 (6)	0.0382 (6)	0.0329 (6)	0.0018 (5)	0.0168 (5)	0.0003 (5)
C2	0.0472 (7)	0.0402 (7)	0.0346 (7)	0.0016 (5)	0.0175 (6)	−0.0010 (5)
C3	0.0649 (9)	0.0500 (8)	0.0325 (7)	0.0041 (7)	0.0213 (6)	0.0012 (6)
C4	0.0663 (9)	0.0480 (8)	0.0462 (8)	0.0050 (7)	0.0341 (7)	0.0081 (6)
C5	0.0460 (7)	0.0439 (7)	0.0491 (8)	0.0041 (6)	0.0262 (6)	0.0052 (6)
C6	0.0526 (8)	0.0497 (8)	0.0706 (10)	−0.0007 (6)	0.0359 (8)	0.0089 (7)
C7	0.0431 (8)	0.0493 (8)	0.0803 (11)	−0.0071 (6)	0.0261 (8)	0.0016 (8)
C8	0.0373 (7)	0.0473 (8)	0.0586 (9)	0.0000 (6)	0.0139 (6)	−0.0044 (6)
C9	0.0379 (7)	0.0440 (7)	0.0437 (7)	0.0003 (5)	0.0162 (6)	−0.0008 (6)
C10	0.0378 (6)	0.0386 (7)	0.0414 (7)	0.0038 (5)	0.0192 (5)	0.0010 (5)
C11	0.0418 (7)	0.0358 (6)	0.0290 (6)	−0.0019 (5)	0.0121 (5)	−0.0027 (5)
C12	0.0415 (7)	0.0390 (6)	0.0263 (6)	−0.0027 (5)	0.0135 (5)	−0.0024 (5)
C13	0.0460 (7)	0.0379 (6)	0.0329 (6)	−0.0023 (5)	0.0158 (5)	−0.0040 (5)
C14	0.0467 (7)	0.0437 (7)	0.0431 (7)	0.0019 (6)	0.0192 (6)	−0.0054 (6)
C15	0.0452 (7)	0.0524 (8)	0.0439 (7)	−0.0053 (6)	0.0209 (6)	−0.0090 (6)
C16	0.0525 (8)	0.0431 (7)	0.0477 (8)	−0.0099 (6)	0.0237 (6)	−0.0031 (6)
C17	0.0471 (7)	0.0386 (7)	0.0374 (7)	−0.0031 (5)	0.0170 (6)	−0.0002 (5)
C18	0.0582 (9)	0.0627 (10)	0.0544 (10)	−0.0011 (8)	0.0014 (8)	0.0022 (7)
C19	0.0629 (10)	0.0782 (12)	0.0547 (10)	−0.0117 (9)	0.0035 (8)	−0.0099 (9)
C20	0.0562 (8)	0.0371 (7)	0.0630 (9)	0.0008 (6)	0.0276 (7)	−0.0007 (6)
C21	0.0526 (9)	0.0698 (10)	0.0793 (12)	−0.0065 (8)	0.0358 (9)	−0.0094 (9)
C22	0.0570 (9)	0.0386 (7)	0.0753 (11)	−0.0005 (6)	0.0243 (8)	0.0049 (7)

### Geometric parameters (Å, °)

**Table:**

O1—C11	1.2201 (15)	C13—C14	1.3916 (19)
O2—C2	1.3696 (16)	C13—C20	1.5112 (18)
O2—C18	1.4190 (18)	C14—C15	1.3858 (19)
O3—C8	1.3650 (18)	C14—H14	0.9500
O3—C19	1.419 (2)	C15—C16	1.383 (2)
C1—C2	1.3840 (18)	C15—C21	1.5087 (19)
C1—C10	1.4296 (18)	C16—C17	1.3924 (19)
C1—C11	1.4991 (16)	C16—H16	0.9500
C2—C3	1.4105 (18)	C17—C22	1.5111 (19)
C3—C4	1.363 (2)	C18—H18A	0.9800
C3—H3	0.9500	C18—H18B	0.9800
C4—C5	1.407 (2)	C18—H18C	0.9800
C4—H4	0.9500	C19—H19A	0.9800
C5—C6	1.420 (2)	C19—H19B	0.9800
C5—C10	1.4236 (18)	C19—H19C	0.9800
C6—C7	1.352 (2)	C20—H20A	0.9800
C6—H6	0.9500	C20—H20B	0.9800
C7—C8	1.412 (2)	C20—H20C	0.9800
C7—H7	0.9500	C21—H21A	0.9800
C8—C9	1.3694 (19)	C21—H21B	0.9800
C9—C10	1.4208 (18)	C21—H21C	0.9800
C9—H9	0.9500	C22—H22A	0.9800
C11—C12	1.4982 (17)	C22—H22B	0.9800
C12—C17	1.4051 (17)	C22—H22C	0.9800
C12—C13	1.4056 (18)		
			
C2—O2—C18	118.01 (11)	C15—C14—H14	118.9
C8—O3—C19	117.79 (12)	C13—C14—H14	118.9
C2—C1—C10	119.75 (11)	C16—C15—C14	118.02 (12)
C2—C1—C11	120.07 (11)	C16—C15—C21	121.52 (13)
C10—C1—C11	120.17 (11)	C14—C15—C21	120.46 (13)
O2—C2—C1	116.54 (11)	C15—C16—C17	122.36 (12)
O2—C2—C3	122.23 (12)	C15—C16—H16	118.8
C1—C2—C3	121.16 (12)	C17—C16—H16	118.8
C4—C3—C2	119.46 (13)	C16—C17—C12	118.59 (12)
C4—C3—H3	120.3	C16—C17—C22	119.09 (12)
C2—C3—H3	120.3	C12—C17—C22	122.28 (12)
C3—C4—C5	121.69 (12)	O2—C18—H18A	109.5
C3—C4—H4	119.2	O2—C18—H18B	109.5
C5—C4—H4	119.2	H18A—C18—H18B	109.5
C4—C5—C6	121.78 (13)	O2—C18—H18C	109.5
C4—C5—C10	119.41 (13)	H18A—C18—H18C	109.5
C6—C5—C10	118.80 (13)	H18B—C18—H18C	109.5
C7—C6—C5	121.20 (13)	O3—C19—H19A	109.5
C7—C6—H6	119.4	O3—C19—H19B	109.5
C5—C6—H6	119.4	H19A—C19—H19B	109.5
C6—C7—C8	120.10 (14)	O3—C19—H19C	109.5
C6—C7—H7	120.0	H19A—C19—H19C	109.5
C8—C7—H7	120.0	H19B—C19—H19C	109.5
O3—C8—C9	124.67 (14)	C13—C20—H20A	109.5
O3—C8—C7	114.41 (13)	C13—C20—H20B	109.5
C9—C8—C7	120.91 (14)	H20A—C20—H20B	109.5
C8—C9—C10	120.13 (13)	C13—C20—H20C	109.5
C8—C9—H9	119.9	H20A—C20—H20C	109.5
C10—C9—H9	119.9	H20B—C20—H20C	109.5
C9—C10—C5	118.84 (12)	C15—C21—H21A	109.5
C9—C10—C1	122.61 (11)	C15—C21—H21B	109.5
C5—C10—C1	118.49 (12)	H21A—C21—H21B	109.5
O1—C11—C12	120.33 (11)	C15—C21—H21C	109.5
O1—C11—C1	119.28 (11)	H21A—C21—H21C	109.5
C12—C11—C1	120.39 (10)	H21B—C21—H21C	109.5
C17—C12—C13	120.11 (12)	C17—C22—H22A	109.5
C17—C12—C11	119.04 (11)	C17—C22—H22B	109.5
C13—C12—C11	120.82 (11)	H22A—C22—H22B	109.5
C14—C13—C12	118.76 (12)	C17—C22—H22C	109.5
C14—C13—C20	118.30 (12)	H22A—C22—H22C	109.5
C12—C13—C20	122.87 (12)	H22B—C22—H22C	109.5
C15—C14—C13	122.15 (12)		
			
C18—O2—C2—C1	161.62 (13)	C11—C1—C10—C9	−4.59 (18)
C18—O2—C2—C3	−21.32 (19)	C2—C1—C10—C5	−0.93 (18)
C10—C1—C2—O2	178.56 (11)	C11—C1—C10—C5	178.15 (11)
C11—C1—C2—O2	−0.52 (17)	C2—C1—C11—O1	130.97 (13)
C10—C1—C2—C3	1.46 (19)	C10—C1—C11—O1	−48.11 (17)
C11—C1—C2—C3	−177.62 (12)	C2—C1—C11—C12	−49.47 (16)
O2—C2—C3—C4	−177.48 (12)	C10—C1—C11—C12	131.46 (12)
C1—C2—C3—C4	−0.5 (2)	O1—C11—C12—C17	−46.12 (17)
C2—C3—C4—C5	−0.9 (2)	C1—C11—C12—C17	134.33 (12)
C3—C4—C5—C6	−177.78 (14)	O1—C11—C12—C13	131.78 (13)
C3—C4—C5—C10	1.4 (2)	C1—C11—C12—C13	−47.77 (16)
C4—C5—C6—C7	178.76 (14)	C17—C12—C13—C14	−1.15 (18)
C10—C5—C6—C7	−0.4 (2)	C11—C12—C13—C14	−179.03 (11)
C5—C6—C7—C8	−0.8 (2)	C17—C12—C13—C20	175.68 (12)
C19—O3—C8—C9	0.5 (2)	C11—C12—C13—C20	−2.20 (18)
C19—O3—C8—C7	−179.03 (14)	C12—C13—C14—C15	0.25 (19)
C6—C7—C8—O3	−179.25 (14)	C20—C13—C14—C15	−176.73 (13)
C6—C7—C8—C9	1.2 (2)	C13—C14—C15—C16	0.8 (2)
O3—C8—C9—C10	−179.74 (12)	C13—C14—C15—C21	−179.45 (13)
C7—C8—C9—C10	−0.2 (2)	C14—C15—C16—C17	−1.0 (2)
C8—C9—C10—C5	−1.05 (19)	C21—C15—C16—C17	179.27 (13)
C8—C9—C10—C1	−178.31 (12)	C15—C16—C17—C12	0.1 (2)
C4—C5—C10—C9	−177.84 (12)	C15—C16—C17—C22	−177.54 (13)
C6—C5—C10—C9	1.38 (19)	C13—C12—C17—C16	0.97 (18)
C4—C5—C10—C1	−0.47 (18)	C11—C12—C17—C16	178.89 (11)
C6—C5—C10—C1	178.75 (12)	C13—C12—C17—C22	178.55 (12)
C2—C1—C10—C9	176.34 (11)	C11—C12—C17—C22	−3.53 (19)

### Hydrogen-bond geometry (Å, °)

**Table:**

*D*—H···*A*	*D*—H	H···*A*	*D*···*A*	*D*—H···*A*
C4—H4···O1^i^	0.95	2.54	3.3756 (18)	147
C7—H7···O2^ii^	0.95	2.60	3.466 (2)	152
C9—H9···O1	0.95	2.39	2.9464 (17)	117
C18—H18B···O3^iii^	0.98	2.59	3.471 (2)	149
C22—H22A···O1	0.98	2.51	2.885 (2)	102

Symmetry codes: (i) *x*, −*y*+1/2, *z*−1/2; (ii) −*x*+1, *y*+1/2, −*z*+3/2;  (iii) *x*−1, −*y*+1/2, *z*−1/2.
